# Pancreatic alpha cell-selective deletion of *Tcf7l2* impairs glucagon secretion and counter-regulatory responses to hypoglycaemia in mice

**DOI:** 10.1007/s00125-017-4242-2

**Published:** 2017-03-25

**Authors:** Gabriela da Silva Xavier, Angeles Mondragon, Vishnou Mourougavelou, Céline Cruciani-Guglielmacci, Jessica Denom, Pedro Luis Herrera, Christophe Magnan, Guy A. Rutter

**Affiliations:** 1 Section of Cell Biology and Functional Genomics, Department of Medicine, Imperial College London, London, W12 0NN UK; 2 Université Paris Diderot Paris 7 - CNRS UMR 8251, Paris, France; 3 Department of Genetic Medicine & Development, Faculty of Medicine, University of Geneva, Geneva, Switzerland

**Keywords:** Alpha cell, Diabetes, Gene, Glucagon, GWAS, Islet

## Abstract

**Aims/hypothesis:**

Transcription factor 7-like 2 (TCF7L2) is a high mobility group (HMG) box-containing transcription factor and downstream effector of the Wnt signalling pathway. SNPs in the *TCF7L2* gene have previously been associated with an increased risk of type 2 diabetes in genome-wide association studies. In animal studies, loss of *Tcf7l2* function is associated with defective islet beta cell function and survival. Here, we explore the role of TCF7L2 in the control of the counter-regulatory response to hypoglycaemia by generating mice with selective deletion of the *Tcf7l2* gene in pancreatic alpha cells.

**Methods:**

Alpha cell-selective deletion of *Tcf7l2* was achieved by crossing mice with floxed *Tcf7l2* alleles to mice bearing a *Cre* recombinase transgene driven by the preproglucagon promoter (PPG*Cre*), resulting in *Tcf7l2*AKO mice. Glucose homeostasis and hormone secretion in vivo and in vitro, and islet cell mass were measured using standard techniques.

**Results:**

While glucose tolerance was unaffected in *Tcf7l2*AKO mice, glucose infusion rates were increased (AUC for glucose during the first 60 min period of hyperinsulinaemic–hypoglycaemic clamp test was increased by 1.98 ± 0.26-fold [*p* < 0.05; *n* = 6] in *Tcf7l2*AKO mice vs wild-type mice) and glucagon secretion tended to be lower (plasma glucagon: 0.40 ± 0.03-fold vs wild-type littermate controls [*p* < 0.01; *n* = 6]). *Tcf7l2*AKO mice displayed reduced fasted plasma glucose concentration. Glucagon release at low glucose was impaired in islets isolated from *Tcf7l2*AKO mice (0.37 ± 0.02-fold vs islets from wild-type littermate control mice [*p* < 0.01; *n* = 6). Alpha cell mass was also reduced (72.3 ± 20.3% [*p* < 0.05; *n* = 7) in *Tcf7l2*AKO mice compared with wild-type mice.

**Conclusions/interpretation:**

The present findings demonstrate an alpha cell-autonomous role for *Tcf7l2* in the control of pancreatic glucagon secretion and the maintenance of alpha cell mass and function.

## Introduction

Personalised treatments for type 2 diabetes are moving closer to reality with the information made available [1] through genome-wide association studies (GWAS) and the technical advances that are making genome sequencing much more affordable. One of the major challenges post-GWAS is in understanding precisely how, at the cellular level, the risk variants contribute to disease risk. Such information is likely to be critical for the rational design of therapies [2]. Frequently, the implicated SNPs occur in non-coding regions in the genome, making it difficult to assess how they lead to disease [2]. A prime example is the intronic SNP rs7903146 in the gene encoding transcription factor 7 like-2 (TCF7L2). Risk allele carriers have a ~1.5-fold higher risk for type 2 diabetes per allele [3–6] and for latent autoimmune diabetes in later life [7]. Individuals carrying the rs7903146 risk allele display defective beta cell function, with evidence of reduced beta cell mass and survival [3–14]. There is a growing body of literature on TCF7L2 in the context of glucose homeostasis and diabetes [1–35], with much effort dedicated to elucidating how the SNP alters TCF7L2 expression and how a change in TCF7L2 content in pancreatic islets affects beta cell function [8, 10–16, 19–21, 26–30, 32, 34, 35]. Existing evidence suggests that reduced levels of TCF7L2 in the beta cell [28] lead to impaired insulin secretion [2]. Thus, although TCF7L2 variants have been proposed in one study to act through the liver [10], both clinical data [4–6, 10, 14, 29] and our own [15, 16, 28] and others’ [11, 20, 21] findings using gene silencing in isolated islets, targeted recombination or expression of dominant-negative TCF7L2 in mice are more consistent with an action largely through the endocrine pancreas. Taken together, the available literature thus points to the SNP leading to a loss of TCF7L2 function in pancreatic islets. This may be due to either the increased expression of a tissue-specific, dominant-negative variant of TCF7L2 [26, 29–33], or a lowering in the expression of a more active isoform, perhaps generated by alternative splicing between exons 13 and 14 and expressed selectively in the beta (and alpha) cell [19, 34, 35].

While extensive efforts have been made by us [15, 16, 28] and others [8, 11, 12, 17–23, 25–27] to examine the role of TCF7L2 in pancreatic beta cell function, little is known about the role of this factor in the other pancreatic islet cell types. Here, we describe the consequences of alpha cell-specific deletion of *Tcf7l2* in the mouse. We chose to use C57BL/6 mice for our study as this mouse strain has been extensively used for the study of glucose homeostasis in the context of the study of diabetes in humans. Our hypothesis is that *Tcf7l2* function in the alpha cell is important for the control of glucagon release and the maintenance of glucose homeostasis.

## Methods

### Materials

Unless otherwise stated all materials were obtained from Sigma (Poole, UK).

### Generation and maintenance of alpha cell-selective *Tcf7l2*-knockout mice

Mice carrying conditional null alleles of *Tcf7l2* (*Tcf7l2* ^fl/fl^) were generated as described in [16] and bred into a C57BL/6 background. *Tcf7l2* ^fl/fl^ mice were crossed with mice expressing *Cre* under the control of the a 0.6 kB fragment of the preproglucagon promoter (PPG*Cre* mice [36]; provided by P. Herrera, University of Geneva, Switzerland), which had been crossed into a C57BL/6 background to generate PPG*Cre*:*Tcf7l2* ^fl/fl^ mice (herein referred to as *Tcf7l2*AKO mice), in which there is deletion of *Tcf7l2* in pancreatic alpha cells and limited expression of *Tcf7l2* in extrapancreatic tissue [36–39]. *Tcf7l2*AKO mice were born at the expected Mendelian ratios and male mice were phenotyped at 8–20 weeks of age. Genotyping was performed by PCR using DNA from ear biopsies. Wild-type littermate control (*Tcf7l2* ^fl/fl^) mice lacked the PPG*Cre* allele. Possession of the latter allele exerted no effects on glucose tolerance or glucagon secretion compared with wild-type mice, as previously reported [38]. All mouse lines were maintained on a C57BL/6 background. Mice were housed in groups of two to five per individually ventilated cage in a pathogen-free facility with 12 h light–dark cycle and were fed ad libitum with a standard mouse chow diet. All in vivo procedures described were performed at the Imperial College Central Biomedical Service and approved by the local ethical committee and UK Home Office according to the Animals (Scientific Procedures) Act 1986 of the UK (PPL 70/7971).

### In vivo physiology

#### IPGTT and insulin tolerance test

Mice fasted for 16 h (with free access to water) were injected intraperitoneally with 1 g glucose/kg, and glucose levels in tail-vein blood were measured with an automatic glucometer (Accuchek Compact Plus; Roche, Burgess Hill, UK) [28]. Insulin tolerance was assessed by i.p. injection of insulin (0.75 U/kg; ActRapid, NovoNordisk, London, UK), which was administered to mice that had been subjected to a 5 h fast. Plasma was collected and centrifuged (2000 *g*, 5 min) in heparin-coated tubes (Microvette; Sarstedt, Leicester, UK) and plasma glucagon and glucagon-like peptide-1 (GLP-1) were assessed by radioimmunoassay (Millipore/Linco, Watford, UK). Hyperinsulinaemic–hypoglycaemic clamp tests were performed by perfusion of insulin and glucose solutions through a jugular catheter, as described [39].

### Islet isolation, in vitro glucose-stimulated glucagon secretion and real-time PCR analysis

After mice were euthanised by cervical dislocation, islets were purified on histopaque gradients and hand-picked as described [40]. Islets were cultured in RPMI medium (Gibco, Paisley, UK) supplemented with 2 mmol/l glutamine, 100 U/ml of penicillin, 100 U/ml of streptomycin and 10% (vol./vol.) heat-inactivated FBS for 24 h. Secretion from islets (10 per condition, size-matched) was measured in 0.5 ml KRB solution containing 3 or 10 mmol/l glucose as described [41, 42].

Total RNA was extracted in Trizol (Invitrogen, Paisley, UK) from 100 mouse islets and real-time PCR analysis of *Gcg* and *Mafb* was conducted as previously described [43].

### Immunohistochemistry

Beta and alpha cell masses were assessed as previously described [43] in pancreases from 20-week-old mice. Briefly, isolated pancreases were fixed in 10% buffered formalin and embedded in paraffin wax within 24 h of removal. Head-to-tail sections (5 μm lengthwise) were cut and incubated overnight at 37°C on superfrost slides. Slides were submerged sequentially in Histochoice followed by decreasing concentrations of industrial methylated spirits for removal of paraffin wax. TCF7L2 protein content in pancreatic alpha cells was assessed by immunohistochemistry (anti-TCF7L2 antibody [SC-8631]; 1:50 dilution; Santa Cruz, Heidelberg, Germany), as per the manufacturer’s instructions. Images were captured on a Zeiss Axio Observer.Z1 Motorised Inverted Widefield Microscope (Zeiss, Cambridge, UK) fitted with a Hamamatsu Flash 4.0 Camera (Hamamatsu Photonics, Welwyn Garden City, UK) using Plan-Apochromat 20×/0.8 M27 air objective (Zeiss) with Colibri.2 LED illumination. Data acquisition was controlled by Zeiss Zen Blue 2012 software configured at a bit depth of 16-bit and binning mode 2 × 2 (Zeiss). Whole-tissue tiled preview scans were obtained using an EC Plan-Neofluar 10x/0.3 Ph1 air objective with phase contrast (Zeiss). Excitation intensities and exposure times were kept constant for all images. Image analysis was performed using Volocity (PerkinElmer, Beaconsfield, UK) and Fiji (https://fiji.sc/, accessed 25 June 2015) [43]. Experimenters were blinded to the group assignment for assessment of islet cell mass.

### Laser capture microdissection and real-time PCR analysis

Laser capture microdissection was performed on pancreatic slices essentially as described [44]. Alpha and beta cells were identified by fluorescent staining as described in the methods for ‘immunohistochemistry’. Cells were extracted from ten pancreatic slices from three separate pancreases from *Tcf7l2*AKO or wild-type littermate control mice, and pooled for RNA extraction. Real-time quantitative PCR was conducted to analyse*Tcf7l2*, *Gcg*, *Ins2* and *Mafb* expression, as previously described [43].

### Statistical analysis

Samples were not randomised. No data, samples or animals were excluded. Data are expressed as means ± SEM. Significance was tested by two sample unpaired or paired Student’s *t* test using Excel (Microsoft, Reading, UK). A value of *p* < 0.05 was considered significant.

## Results

### Generation of mice deleted for *Tcf7l2* selectively in the pancreatic alpha cell

**Fig. 1:**
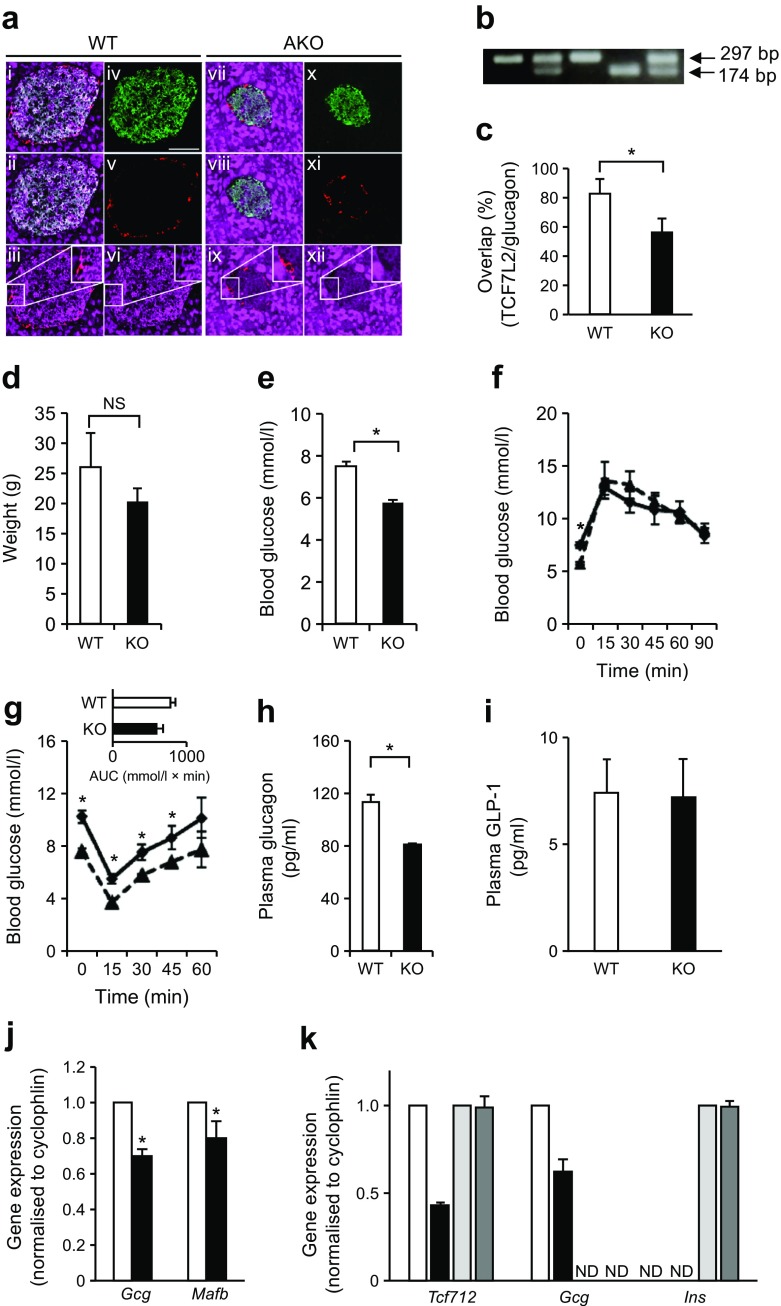
*Tcf7l2*AKO mice display reduced blood glucose, insulin intolerance and plasma glucagon concentration. *Tcf7l2* was knocked out using an alpha cell-selective *Cre* [36]. (**a**) Representative images of immunohistochemical analysis are shown to confirm knockout by labelling pancreases from wild-type (WT; panels i–vi) and *Tcf7l2*AKO (panels vii–xii) mice with anti-glucagon (red), anti-insulin (green) and anti-TCF7L2 (magenta) antibodies. Images i and vii show overlay of all three channels, ii and viii show overlay of insulin with TCF7L2, iii and ix show overlay of glucagon with TCF7L2, and iv–vi and x–xii show the individual channels. Inset panels show magnified images of the indicated areas. Scale bar, 50 μm and applies to all micrographs in part (**a**). (**b**) PCR genotyping gel to confirm the presence of the wild-type (WT; 174 bp) and conditional knockout (AKO; 297 bp) allele. (**c**) Graph showing quantification of the degree of overlap between glucagon-positive alpha cells and TCF7L2-positive cells in pancreases from WT and *Tcf7l2*AKO mice. (**d**) *Tcf7l2*AKO mice exhibit normal weight. (**e**–**g**) i.p. glucose and insulin tolerance tests were conducted on 8–9-week-old mice on a normal chow diet. (**e**) Fasting glucose but not (**f**) overall glucose tolerance was altered in *Tcf7l2*AKO mice compared with WT mice. (**g**) Insulin tolerance, (**h**) fasting (16 h) plasma glucagon and (**i**) plasma GLP-1 were also measured in *Tcf7l2*AKO mice. (**j**) Real-time PCR analysis of islets of Langerhans from 20-week-old *Tcf7l2*AKO mice and WT littermate control mice on normal chow diet. (**k**) Real-time quantitative PCR analysis of cells captured by laser microdissection for measurements of the indicated genes. In (**a–j**): white bars and solid lines, WT mice; black bars and dashed lines, *Tcf7l2*AKO mice. In (**k**): white bars, glucagon-positive cells from WT mice; black bars, glucagon-positive cells from *Tcf7l2*AKO mice; light grey bars, insulin-positive cells from WT mice; dark grey bars, insulin-positive cells from *Tcf7l2*AKO mice. For (**a**–**i**), *n* = 5; for (**j**) and (**k**), *n* = 3. ND, non-detectable (i.e. *Gcg* and *Ins* expression was undetectable in insulin- and glucagon-positive cells, respectively), NS, non-significant. *p < 0.05

Cross-breeding of mice with floxed *Tcf7l2* alleles with mice expressing *Cre* recombinase under the control of the preproglucagon (PPG) gene promoter [36] was predicted to lead to recombination selectively in pancreatic islet alpha cells (generating *Tcf7l2*AKO mice). We used immunohistochemistry to assess TCF7L2 protein content in alpha cells because of the low abundance of alpha cells in rodent islets (~20% of all cells) [45, 46], and expected 20–50% deletion with the PPG*Cre* used here [46]. Correspondingly, immunohistochemical analysis revealed a 56.7 ± 9.5% overlap (vs 83.4 ± 10.6% in pancreases from wild-type littermate control mice) of signal from anti-TCF7L2 antibody with the signal from glucagon in *Tcf7l2*AKO islets (Fig. 1a, c).

### Pancreatic alpha cell-selective deletion of *Tcf7l2* leads to reduced fasting glucose, normal insulin tolerance but impaired counter-regulatory response

There were no significant differences in weight between *Tcf7l2*AKO mice and wild-type littermate control mice (Fig. 1d). Fasting plasma glucose (measured at 09:00 hours following a 16 h fast, Fig. 1e, f) was lower in *Tcf7l2*AKO mice compared with wild-type littermate control mice, while glucose tolerance (as assessed by IPGTT) was unaffected (Fig. 1f). *Tcf7l2*AKO mice exhibited normal tolerance to i.p insulin (Fig. 1g). While plasma glucose levels were significantly higher at all except one time point sampled during the period of the test, there was no significant difference between genotypes in the area under the curve (AUC) for the period of the test (Fig. 1g, inset). *Tcf7l2*AKO mice exhibited reduced fasting plasma glucagon levels (Fig. 1h), suggestive of defective counter-regulatory responses. Plasma GLP-1 levels were unchanged (Fig. 1i), while islet glucagon (*Gcg*) and *Mafb* gene expression were significantly reduced in islets from *Tcf7l2*AKO vs wild-type littermate control mice (Fig. 1j). Gene expression analysis of pooled glucagon-positive cells captured by laser capture microdissection demonstrated a 56.8 ± 5.45% and 37.2 ± 6.52% decrease in *Tcf7l2* and *Gcg* gene expression, respectively, in cells from *Tcf7l2*AKO vs wild-type littermate control mice (Fig. 1k). In contrast, neither *Tcf7l2* nor *Ins* gene expression were significantly affected in insulin-positive cells captured by laser capture microdissection (Fig. 1k).

**Fig. 2:**
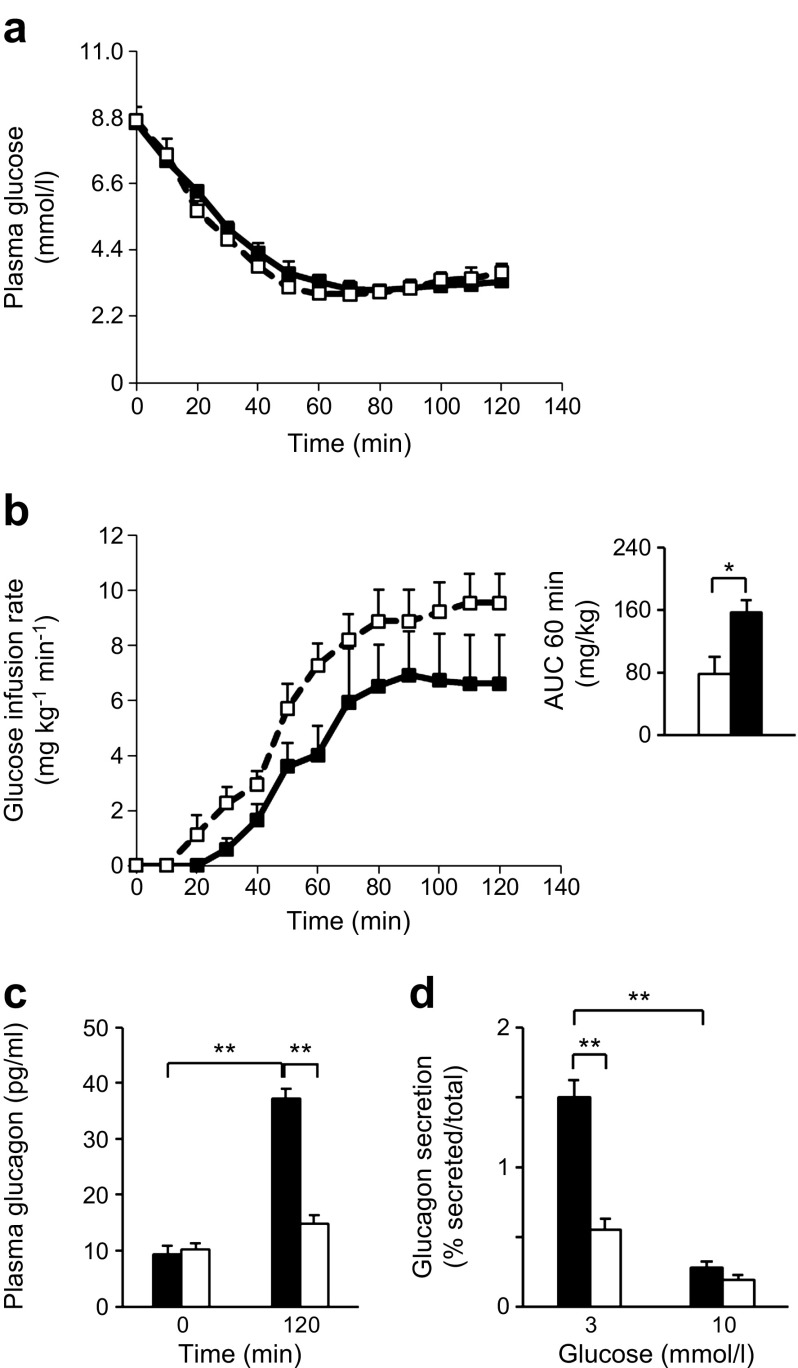
*Tcf7l2*AKO mice display impaired counter-regulatory response to hypoglycaemia. (**a**) Plasma glucose concentration and (**b**) glucose infusion rates for hyperinsulinaemic–hypoglycaemic clamp tests in 20-week-old wild-type (solid line/black squares and black bars) and *Tcf7l2*AKO (dashed lines/white squares and white bars) mice. Insulin was infused at time 0 min; inset in (**b**) shows AUC for glucose infusion rates. (**c**) Plasma glucagon levels in mice undergoing hyperinsulinaemic–hypoglycaemic clamp tests at 0 and 120 min of the glucose infusion protocol. (**d**) Levels of glucagon secretion from isolated islets of Langerhans exposed to glucose at the indicated concentrations. Black bars, wild-type mice; white bars, *Tcf7l2*AKO mice. *n* = 6 mice for all;*p < 0.05 and **p < 0.01

To determine whether the above changes result in impaired glucagon release in vivo we performed hyperinsulinaemic–hypoglycaemic clamp tests. During the time period 20–120 min after the start of insulin infusion, when blood glucose levels were similar in both groups (Fig. 2a), it was necessary to infuse glucose more rapidly into *Tcf7l2*AKO vs wild-type littermate control mice to maintain blood glucose levels (Fig. 2b). Thus, a significant increase (1.98 ± 0.26-fold; *p* < 0.05) in the AUC for glucose was observed during the first 60 min period (Fig. 2b [inset]*)*, and plasma glucagon was reduced at 120 min (0.40 ± 0.03 fold; *p* < 0.01; Fig. 2c) vs wild-type littermate control mice following glucose infusion, confirming a defective counter-regulatory response in these mice. Likewise, glucagon secretion from isolated islets was significantly reduced in response to low glucose (3 mmol/l; Fig. 2d), while total islet glucagon content was not significantly different between *Tcf7l2*AKO vs islets from wild-type littermate control mice (12.1 ± 0.8 vs 13.6 ± 0.9 ng per ten islets, respectively).

### Pancreatic alpha cell mass is reduced in *Tcf7l2*AKO mice

**Fig. 3:**
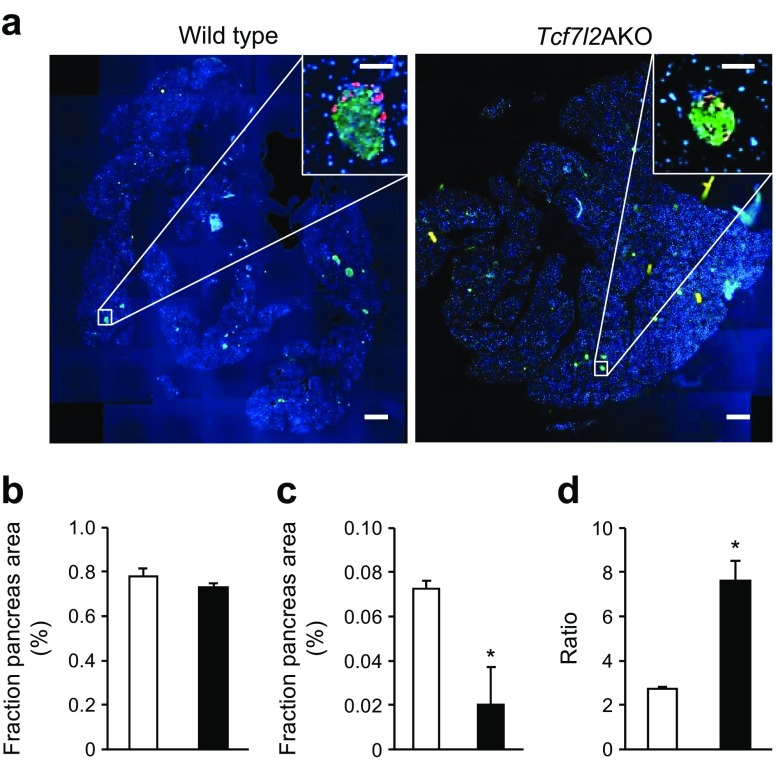
*Tcf7l2*AKO mice display reduced alpha cell mass. (**a**) Representative images from pancreatic sections from wild-type and *Tcf7l2*AKO mice are shown. Scale bar in large micrographs, 500 μm; scale bar in insets, 100 μm. (**b**) Beta cell (defined as insulin-positive cells, in green) and (**c**) alpha cell (defined as glucagon-positive cells, in red) mass, and (**d**) beta/alpha cell ratio from 20-week-old mice were quantified [44]. White bars, wild-type mice; black bars, *Tcf7l2*AKO mice. *n* = 7 mice for all; *p < 0.05

Immunohistochemical analysis revealed no change in beta cell mass (Fig. 3b) but a 72.3 ± 20.3% (*p* < 0.05) decrease in alpha cell mass in *Tcf7l2*AKO vs wild-type littermate control mice (Fig. 3c), resulting in a 2.8 ± 0.09-fold increase in beta/alpha cell mass ratio (Fig. 3d).

## Discussion

It was recently demonstrated that individuals without diabetes bearing rs7903146 risk variants display an increased pancreatic alpha/beta cell ratio [10]. We [15, 16, 28] and others [8, 11, 12, 17–23, 25–27], have previously demonstrated that loss of *Tcf7l2* from pancreatic islets [16] or selectively from beta cells [28] leads to decreased beta cell mass and increased beta cell apoptosis [11–13, 16, 21, 28, 29, 32, 47]. The observation that alpha cell mass was not altered in mice with beta cell-specific deletions in *Tcf7l2* and glucose intolerance [28] suggests that the increase in this variable in risk allele carriers may be due to a cell-autonomous role for TCF7L2 in the alpha cell.

As a means of testing this hypothesis directly, we provide here the first description of mice with pancreatic alpha cell-specific deletion of *Tcf7l2*. *Tcf7l2*AKO mice present with a robust reduction of TCF7L2 protein content (Fig. 1a, c) and *Tcf7l2* expression (Fig. 1k) in alpha cells, reflecting the expected efficiency of the PPG*Cre* strain (which recombines in 20–50% of alpha cells; see [39] and references therein). In addition, we demonstrated that the expression of two alpha cell-specific genes, *Gcg* and *Mafb*, was reduced in pancreatic islets (Fig. 1j), although interestingly these changes did not result in an apparent lowering of islet glucagon content (described further below). The reason(s) for this discordance between changes at the mRNA and protein level are unclear.

We note that although other *Cre* driver lines under the control of the glucagon promoter result in more complete recombination in alpha cells [48, 49]. However, these also recombine in the brain and in intestinal L cells, complicating the interpretation of results obtained through their use. This is particularly relevant as TCF7L2 has recently been shown to regulate gut and brain proglucagon gene expression and glucose homeostasis [22]. However, unlike the *Gcg* promoter used in the Shao study [22], the PPG*Cre* strain we use in this study has previously been shown to exhibit no recombination in the brain and <5% recombination in the small intestine, with no effect on plasma GLP-1 content [37]. Here, we show that plasma GLP-1 levels were unchanged (Fig. 1i) in *Tcf7l2*AKO mice in comparison with wild-type littermate controls, indicating that deletion of *Tcf7l2* in cells in which the PPG promoter is active did not significantly alter GLP-1 production or release.

One of our key findings was that 8–9-week-old *Tcf7l2*AKO mice have lower fasted (Fig. 1e, f) blood glucose concentrations. While glucose (Fig. 1f) and insulin tolerance (Fig. 1g) was unaffected in *Tcf7l2*AKO mice, *Tcf7l2*AKO mice displayed lower fasting plasma glucagon (Fig. 1h) compared with wild-type littermate controls. These data point to a defective counter-regulatory response (where, in health, an increase in glucagon release from pancreatic alpha cells leads to avoidance of hypoglycaemia) which we confirmed by performing hyperinsulinaemic–hypoglycaemic clamps (Fig. 2). Glucagon secretion from isolated islets in response to low glucose levels (3 mmol/l) was similarly impaired (Fig. 2d), while islet glucagon content was unaltered in *Tcf7l2*AKO vs islets from wild-type littermate control mice, and alpha cell mass was decreased (Fig. 3). Thus, the loss of counter-regulatory response in *Tcf7l2*AKO mice would appear to reflect, at least in large part, decreases in both alpha cell mass and function. The downstream targets of *Tcf7l2* that may mediate the molecular mechanisms that lead to the loss of alpha cell mass and function remain to be determined.

The present findings thus extend our published data on *Tcf7l2* function in pancreatic beta cells [28], indicating that TCF7L2 has a cell-autonomous function in pancreatic islet alpha cells through modulation of cell mass, function and the expression of *Gcg* and other genes in these cells. The molecular mechanisms that underlie these changes remain, however, to be characterised in detail, though actions on membrane potential or calcium dynamics, as reported in beta cells deficient in *Tcf7l2* [15], are likely possibilities.

Intriguingly, the present and previous [14] data indicate that loss of TCF7L2 function, as anticipated in carriers of the risk allele rs7903146 as a result of increases in the expression of dominant-negative isoforms of the protein, leads to decreases in both alpha and beta [28] cell mass. The combined effect is an overall larger impact on islet function, potentially having an impact on pathways that are involved in islet cell regeneration in disease conditions. Importantly a decrease of similar magnitude in the functional mass of both cell types is expected to reduce antihyperglycaemic drives postprandially, when alpha cells are largely inactive in individuals without diabetes, thus increasing diabetes risk. Data are currently unavailable on the effect of risk alleles on the expression of *Tcf7l2* mRNA levels and splicing in alpha cells. Of note, we have previously hypothesised that risk variants may not act on TCF7L2 activity in the liver if the affected splice variant containing exons 13, 13b and 14 is not present in this tissue [19, 28, 34, 35, 50]. Studies on purified human alpha cell populations will be required to address this question.

Interestingly, we have also recently shown [39] that loss of the secretory granule zinc transporter ZnT8, encoded by the type 2 diabetes GWAS gene *SLC30A8* [5] from the murine alpha cell, leads to exaggerated glucagon release in response to low glucose levels, though alpha cell mass was not altered in the latter model. Conversely, overexpression of ZnT8 in alpha cells stimulates glucagon secretion [51]. The latter findings demonstrate that the increased abundance of type 2 diabetes in risk allele carriers might, in both cases, involve alterations in glucagon release, consistent with the ‘dual hormone’ model for this disease [52]. Whether personalised treatments of type 2 diabetes based on genotype at either locus may usefully target these changes in glucagon release may be worthy of exploration in the future. Likewise, variation in the association SNPs in both alleles might be useful in the context of type 1 diabetes as a predictor of counter-regulatory responses to hypoglycaemia.

## Abbreviations

GLP-1: Glucagon-like peptide-1
GWAS: Genome-wide association studies
PPG: Preproglucagon
TCF7L2: Transcription factor 7 like-2
*Tcf7l2*^fl/fl^: Mice carrying conditional null alleles of *Tcf7l2*
*Tcf7l2*AKO: Alpha cell-specific deletion of *Tcf7l2* in the mouse
PPG*Cre*: *Cre* under the control of the a 0.6 kB fragment of the preproglucagon promoter
